# Dynamic and visual nomograms to online predict unfavorable outcome of mechanical thrombectomy for acute basilar artery occlusion

**DOI:** 10.1002/brb3.3297

**Published:** 2023-11-13

**Authors:** Xiding Pan, Shiteng Lin, Liang Xiang, Feng Zhou, Mengyi Xu, Qiong Jie, Zhihong Zhao, Chen Chen, Junshan Zhou, Jianjun Zou

**Affiliations:** ^1^ Department of Pharmacy Nanjing First Hospital, Nanjing Medical University Nanjing P. R. China; ^2^ Department of Neurology Nanjing First Hospital, Nanjing Medical University Nanjing P. R. China; ^3^ School of Basic Medicine and Clinical Pharmacy China Pharmaceutical University Nanjing P. R. China; ^4^ Department of Pharmacy, Women and Children's Hospital, School of Medicine Xiamen University Xiamen P. R. China; ^5^ Department of Neurology The First Affiliated Hospital (People's Hospital of Hunan Province), Hunan Normal University Changsha P. R. China

**Keywords:** basilar artery occlusion, mechanical thrombectomy, nomogram, unfavorable outcome

## Abstract

**Background:**

The evidence of mechanical thrombectomy (MT) in basilar artery occlusion (BAO) was limited. This study aimed to develop dynamic and visual nomogram models to predict the unfavorable outcome of MT in BAO online.

**Methods:**

BAO patients treated with MT were screened. Preoperative and postoperative nomogram models were developed based on clinical parameters and imaging features. An independent dataset was collected to perform external validation. Web‐based calculators were constructed to provide convenient access.

**Results:**

A total of 127 patients were included in the study, and 117 of them were eventually included in the analysis. The nomogram models showed robust discrimination, with an area under the receiver operating characteristic (ROC) of 0.841 (preoperative) and 0.916 (postoperative). The calibration curves showed good agreement. The preoperative predictors of an unfavorable outcome were previous stroke, the National Institutes of Health Stroke Scale (NIHSS) at admission, and the posterior circulation Alberta Stroke Program Early Computed Tomography Score (pc‐ASPECTS). The postoperative predictors were previous stroke, NIHSS at 24 h, and pc‐ASPECTS.

**Conclusion:**

Dynamic and visual nomograms were constructed and validated for the first time for BAO patients treated with MT, which provided precise predictions for the risk of an unfavorable outcome. The preoperative model may assist clinicians in selecting eligible patients, and the postoperative model may facilitate individualized poststroke management.

## Graphical abstract

Dynamic and visual nomograms were constructed and validated for the first time for acute basilar artery occlusion patients treated with MT, which provided precise predictions for the risk of unfavorable outcome.

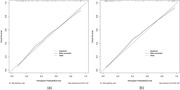

## 1 INTRODUCTION

Globally, China has the highest estimated lifetime risk of stroke in 25 years and beyond. For adults aged 40 years or older, the estimated prevalence and mortality rate of stroke were 2.6%, and 343.4 per 100,000 person‐years, respectively (Tu et al., 2023). Basilar artery occlusion (BAO) accounts for approximately 1% of all ischemic strokes (Schonewille et al., 2009). Despite thrombolytic therapy, the rate of disability is still up to 75% (Sairanen et al., 2011). Mechanical thrombectomy (MT) is an effective recanalization strategy for acute anterior circulation stroke (ACS) caused by large‐vessel occlusion and was recommended by current clinical practice guidelines (Powers et al., 2018). However, for posterior circulation stroke (PCS), the evidence of MT was limited. Two previously published randomized controlled trials (RCT) that investigated the efficacy and safety of MT in PCS (the BEST study and the BASICS study), both failed to prove the superiority of MT over standard medical treatment (Langezaal et al., 2021; Liu et al., 2020). However, the subsequent pooled analysis of these two RCTs (VERITAS study [Nogueira et al., 2021]) and the latest published studies (BAOCHE study [Jovin et al., 2022] and ATTENTION study [Tao et al., 2022]), which focused on patients with NIHSS ≥10, showed the significant efficacy of MT in BAO.

Early prediction of outcomes in BAO patients treated with MT could identify the appropriate patient for MT and provide individual management after stroke. Previous studies have revealed that some factors, such as stroke severity and the success of the operation, were associated with the functional outcome of MT in BAO (Kwak & Park, 2020; Yao et al., 2021; Zhang et al., 2019). So far, no prediction models have been developed to conduct a comprehensive evaluation. A nomogram is a graphical statistical model that is based on diverse clinical and radiological variables and is commonly used to estimate disease prognosis. Its user‐friendly graphical interface facilitates the decision‐making process.

This study aims to develop separate preoperative and postoperative nomogram models to predict the risk of unfavorable outcomes of MT in BAO patients.

## 2 MATERIALS & METHODS

### 2.1 Study population

From October 2016 to June 2021, consecutive BAO patients treated with MT were enrolled in the National Advanced Stroke Center of Nanjing First Hospital (China). The inclusion criteria were: (1) basilar‐artery occlusion was confirmed on computed tomographic angiography, magnetic resonance angiography, or digital subtraction angiography. (2) Within 24 h of symptom onset; (2) 18≤ age ≤80 years; (3) NIHSS score ≥ 6; (4) PC‐ASPECTS score ≥ 6. (5) Patients eligible for intravenous r‐tPA should receive r‐tPA even if endovascular treatments are being considered. The exclusion criteria were: (1) prestroke with a modified Ranking Scale (mRS) > 2; (2) intracranial hemorrhage on neuroimaging; (3) loss to follow‐up; (4) unavailable baseline data; and (5) patients with concomitant anterior circulation occlusion. The present research was approved by the ethics committee of Nanjing First Hospital (document number: KY20130424‐01). The written informed consent was obtained from each participant.

### 2.2 Clinical and radiological variables

Clinical and radiological variables were retrospectively collected from each patient. Demographic data included age, sex, body mass index, and culture. Comorbidities included hypertension, diabetes mellitus, dyslipidemia, coronary artery disease, and a previous history of stroke. Smoking status was divided into three groups: never smoked, former smoker, and current smoker. Similarly, drinking was divided into three groups: never drunk, former drinker, and current drinker. The etiology of stroke was classified according to the TOAST definitions (Adams et al., 1993). Postoperative complications included respiratory infections, secondary epilepsy, gastrointestinal bleeding, and electrolyte disorders. Other data included symptomatic intracranial hemorrhage (sICH), premorbid mRS, National Institutes of Health Stroke Scale (NIHSS) at admission, NIHSS at 24 h, posterior circulation Alberta Stroke Program Early Computed Tomography Score (pc‐ASPECTS), Thrombolysis in Cerebral Infarction score (TICI), prior intravenous thrombolysis, number of retrievals, and other biochemical indicators. We recorded blood pressure variability parameters 1, 3, 6, 12, and 24 h after MT. Then, the standard deviation (SD) and coefficient of variation (CV) of systolic and diastolic blood pressure were calculated using data of these five time points. In addition, we collected the following five procedure time intervals: onset to emergency (OTE), onset to image (OTI), onset to puncture (OTP), onset to recanalization (OTR), and puncture to recanalization (PTR).

mRS at 90 days was collected by telephone or in direct contact with the patient. There were two ways. One was a face‐to‐face interview when the patient consulted in our stroke clinic. Another was the telephone interview. We keep the phone numbers of both patients and their caregivers to ensure that information can be obtained. Unfavorable functional outcome was defined as mRS 4–6. Two independent neurologists assessed angiographic results.

### 2.3 Statistical analysis

The continuous variables were presented by mean (SD) or median (interquartile range). The categorical variables were presented by n (%). The normality of continuous variables was checked by the Shapiro–Wilk test. The comparison of the two groups was performed by the Mann‐Whitney *U* test, *t*‐test, Pearson's chi‐square test, or Fisher's exact test, where appropriate. All tests were two‐sided, and *p*‐values < .05 were considered statistically significant.

### 2.4 Development and assessment of the models

In this study, we first created two multivariable models. The preoperative model incorporated all preoperative variables with a *p*‐value < .05 at univariable analysis. The postoperative model incorporated all the variables (pre‐, intra‐, and postoperative) with *p*‐values < .05 in univariable analysis. The logistic regression method was used for multivariable analysis, with a 90‐day unfavorable outcome as the dependent variable. The collinearity of combinations of variables that entered the multivariable models was evaluated based on variance inflation factors  (<2 were considered nonsignificant). Then, the potential predictors selected by the two multivariable models were used to construct the preoperative and postoperative nomogram models, respectively.

The discrimination accuracy and the calibration of nomogram models were assessed using receiver operating characteristic (ROC) curves and calibration curves. The asymptotically exact method described by DeLong et al. (1988) was used to compare the ROC curves of the pre‐ and postoperative models. Calibration plots were derived with 1000 bootstrap resamplings to describe the relevance of the actual unfavorable outcome to the predicted probability of the unfavorable outcome. All statistical analyses were computed using SPSS 25.0 (IBM Corporation) and R software (R Foundation for Statistical Computing).

### 2.5 External validation and web‐based calculators

The external validation was performed to evaluate the potential and applicability of our nomogram models. The independent dataset of the external validation cohort was collected in People's Hospital of Hunan Province from February 2021 to March 2022. ROC curves were used to evaluate the prediction performance. The “DynNom” package of R software was used to build the dynamic nomogram models (Jalali et al., 2019). Then, web‐based nomogram models were constructed to facilitate online access.

## 3 RESULTS

### 3.1 Study population

**FIGURE 1:**
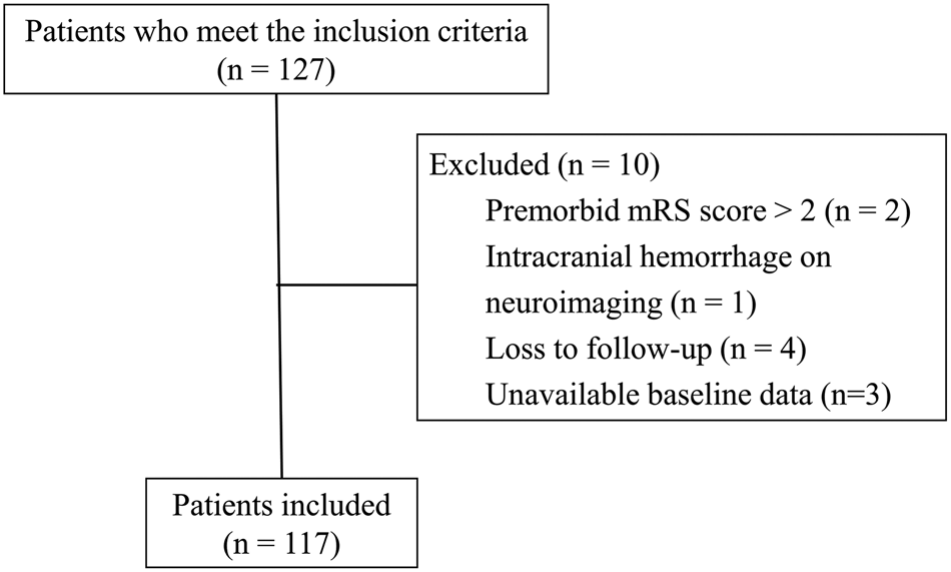
Flow chart demonstrating the number (n) of patients included in the analysis.

A total of 127 patients were included in the study, and 117 of them were eventually included in the analysis (Figure 1). Among them, 97 patients were in the model development group, and another 20 patients from the People's Hospital of Hunan Province were in the external validation group. The unfavorable outcome was observed in 46 (47.4%) patients at 90 days. The median age of overall patients was 68 years (interquartile range: 61–76). A total of 75 (77.3%) patients were males. The clinical and radiological characteristics of the whole cohort (n = 97), the favorable outcome cohorts (n = 51), and the unfavorable outcome (n = 46) cohorts are listed in Supporting Information Table S1.

### 3.2 Feature selection

After univariable analysis, five preoperative and three postoperative variables significantly differed between the two groups among the 42 included variables (Supporting Information Table S1). The five preoperative variables were previous stroke, drinking, NIHSS at admission, pc‐ASPECTS, and International Normalized Ratio. The three postoperative variables were sICH, NIHSS at 24 h, and electrolyte disorders. Then, the selected preoperative variables and all variables were entered into two separate multivariable models. Finally, three variables: previous stroke (odds ratio [OR]: 6.79, 95% confidence of interval [CI]: 1.94–23.74), NIHSS at admission (OR: 1.09, 95% CI: 1.04–1.14), and pc‐ASPECTS (OR: 0.63, 95% CI: 0.46–0.87) in the first multivariable analysis were availed to construct the preoperative nomogram model (Table 1). In the second multivariable analysis, previous stroke (OR: 6.64, 95% CI: 1.52–29.06), NIHSS at 24 h (OR: 1.14, 95% CI: 1.09–1.20), and pc‐ASPECTS (OR: 0.63, 95% CI: 0.44–0.92) were incorporated into the logistic regression model to build the postoperative nomogram model (Table 2).

**TABLE 1:** Multivariable logistic regression analysis based on preoperative data.

Variable	OR	95% CI	*p*‐Value
Previous stroke	6.79	1.94–23.74	.003
NIHSS at admission	1.09	1.04–1.14	<.001
pc‐ASPECTS	0.63	0.46–0.87	.004

NIHSS, National Institutes of Health Stroke Scale; pc‐ASPECTS, posterior circulation Alberta Stroke Program Early Computed Tomography Score.

**TABLE 2:** Multivariable logistic regression analysis based on all collected data.

Variable	OR	95% CI	*p*‐Value
Previous stroke	6.64	1.52–29.06	.012
NIHSS at 24h	1.14	1.09–1.20	<.001
pc‐ASPECTS	0.63	0.44–0.92	.016

NIHSS, National Institutes of Health Stroke Scale; pc‐ASPECTS, posterior circulation Alberta Stroke Program Early Computed Tomography Score.

### 3.3 Development and assessment of the models

**FIGURE 2:**
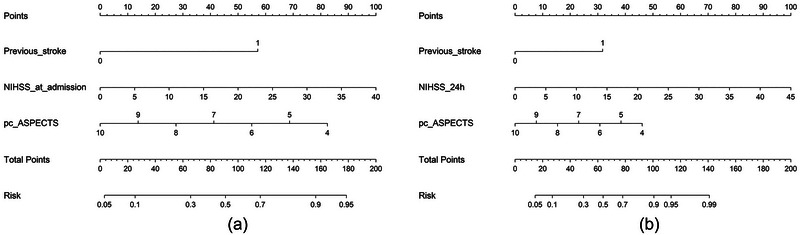
Preoperative (a) and postoperative (b) nomogram to predict unfavorable outcome (modified Rankin Scale 4–6) at 90 days.

The pre‐ and postoperative nomograms based on the two multivariable models are shown in Figure 2a,b. Each factor in the nomograms was assigned a weighted score. The weighted points of each variable were added together to calculate the total points. The corresponding unfavorable outcome risk can be obtained according to Figure 2.

**FIGURE 3:**
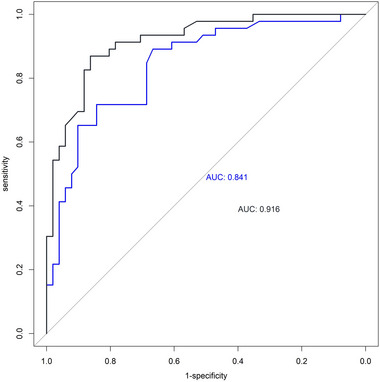
The receiver operating characteristic curves (ROC) of the pre‐ and postoperative nomogram model.

The predictive performance was observed in the preoperative nomogram (area under curve [AUC]: 0.841; 95% CL, 0.761–0.920); and postoperative nomogram (AUC: 0.916; 95% CL: 0.862–0.971) in our dataset (Figure 3). Based on the DeLong test, the postoperative nomogram indicated a significantly higher AUC than the preoperative (DeLong test, *Z* = −2.6187, *p*‐value = .0088).

**FIGURE 4:**
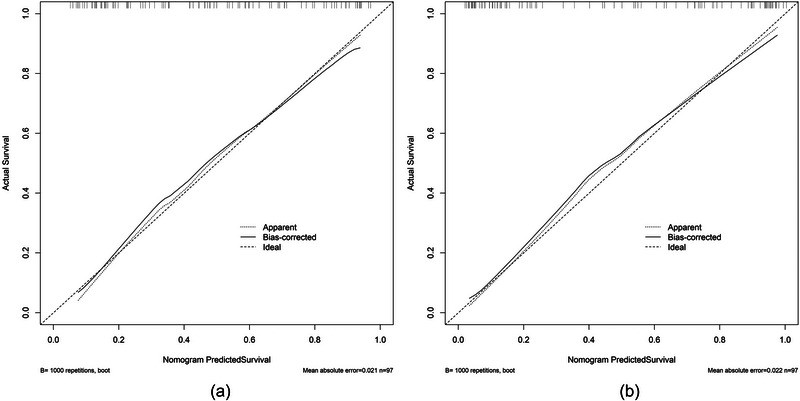
Calibration plots of the preoperative (a) and postoperative (b) nomogram model.

Both in the pre‐ and postoperative nomogram, the points of the calibration plot for the probability of unfavorable outcome in BAO patients were close to the 45° line, which demonstrates positive compliance between prediction of the nomogram and the actual observation (Figure 4a,b). In addition, the mean squared error of the postoperative model was lower than that of the preoperative model, which means the calibration performance was better in the postoperative model.

### 3.4 External validation and web‐based calculators

**FIGURE 5:**
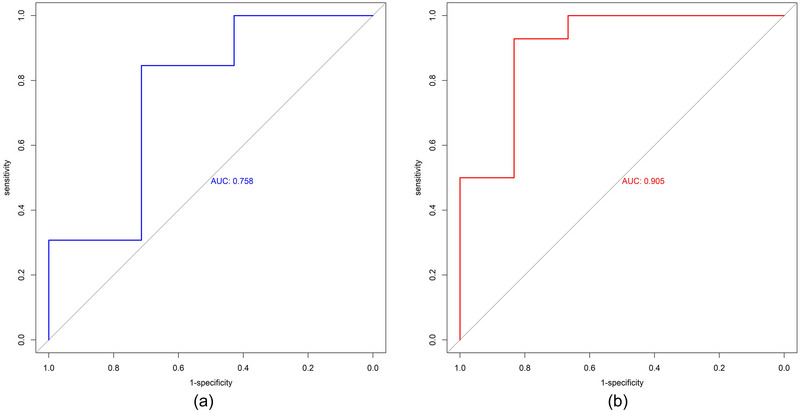
The receiver operating characteristic curves (ROC) of the preoperative (a) and postoperative (b) nomogram model on the external validation cohort.

A total of 20 patients from the independent dataset were included in the external validation cohort. In the external validation cohort, the predictive performance (AUC) of pre‐ and postoperative nomogram were 0.758 and 0.905, respectively (Figure 5).

We have developed two web‐based calculators to facilitate the use of the models (https://trend.shinyapps.io/Pre‐model/ and https://trend.shinyapps.io/post‐model/)

## 4 DISCUSSION

In this study, the dynamic and visual nomograms were constructed and validated for the first time in BAO patients treated with MT. The major strength of this study is that our models demonstrated favorable predictive and calibration capabilities. Besides, the three variables in the models are acquirable, and the dynamic visualized models on the webpage are convenient to use. The preoperative model aimed to assist the clinician in selecting eligible patients for MT, and the postoperative model was more accurate for outcome prediction.

We provide two examples to help clinicians and researchers better understand the utility of network‐based nomograms. For example, if the patient had no history of stroke, the NIHSS at admission is 16, the pc‐ASPECTS is 8, then in the preoperative model, the risk of a 90‐day unfavorable outcome is 0.314 (95% CL, 0.201–0.453) (Supporting Information Figure S1), which may be considered as an appropriate candidate for MT. If a patient had a history of stroke, NIHSS at 24 h is 20, and pc‐ASPECTS is 8, in the postoperative model, the risk of a 90‐day unfavorable outcome risk is 0.839 (95% CL, 0.582–0.952) (Supporting Information Figure S2), which means the intensive care was needed after MT. Certainly, we need to emphasize that the prediction of our nomogram models is only a reference, but not compulsory.

So far, several studies have evaluated the prognostic factors of MT in BAO. They all used multivariable logistical regression models to ascertain the potential predictors (Alexandre et al., 2021; Bouslama et al., 2017; Diprose et al., 2020; Fabritius et al., 2021; Guenego et al., 2021; Kwak & Park, 2020; Mokin et al., 2016; Sang et al., 2021; Wu et al., 2021; Wu, Xu et al., 2021; Yao et al., 2021). In this study, we constructed separate pre‐ and postoperative nomogram models to conduct a comprehensive evaluation. In our study, approximately 47.4% of BAO patients had unfavorable outcome (mRS: 4–6), which is lower than the BEST study (Liu et al., 2020)(67%),and BASICS study (Tu et al., 2023) (64.9%). The possible explanation is that in our nonrandomized observational study, the most severe cases prone to unsatisfied results were excluded from MT after the evaluation. In our study, previous stroke, NIHSS at admission, and pc‐ASPECTS were the preoperative predictors of the unfavorable outcome of BAO patients with MT. The postoperative predictors were the previous stroke, NIHSS at 24 h, and pc‐ASPECTS.

This study was the first to demonstrate that previous stroke strongly predicted the unfavorable outcome of MT. Previous stroke was a major risk factor for recurrent stroke. The cumulative incidence of clinical recurrent stroke was 5.4% at 1 year and 11.3% at 5 years (Khanevski et al., 2019). Patients with previous stroke were usually older and had more comorbidities such as hypertension, diabetes, and chronic kidney disease (Leker et al., 2019). This population was at a higher risk of atherosclerosis. In our study, the most common cause of BAO was large‐artery atherosclerosis (LAA) (67%). It was consistent with the BEST study (56%), which included only the Chinese population. However, in the studies including the Western people, the proportion of LAA patients dropped to about 31—35% (Alexandre et al., 2021; Bouslama et al., 2017). This finding indicates that ethnic differences should be considered in future studies.

Consistent with previous studies, this study confirmed that the pc‐ASPECTS score was correlated with MT outcome (Alemseged et al., 2019; Sang et al., 2021; Wu et al., 2021; Yao et al., 2021). A previous study with a large sample size revealed that patients with pc‐ASPECTS ≥5 could benefit from MT (Sang et al., 2021), while another small sample study showed pc‐ASPECTS ≥8 was the boundary (Alemseged et al., 2019; Fabritius et al., 2021). Furthermore, in patients with pc‐ASPECTS <8, the clinical outcome seemed highly dependent on the rapidity of successful reperfusion (Guillaume et al., 2019). Besides the scores, it is noteworthy that pc‐ASPECTS does not reflect the location or volume of the lesion. Thus, even with the same score, the patient with a critical site occlusion (such as mesencephalon and diencephalon) may have a more devastating outcome.

The association between a high NIHSS score and the risk of an unfavorable outcome was fully demonstrated (Alexandre et al., 2021; Bouslama et al., 2017; Kwak & Park, 2020; Szmygin et al., 2021; Wu et al., 2021; Yao et al., 2021). In the studies of ACS, the baseline NIHSS was approximately 17 (Campbell et al., 2015). However, in BAO studies, the baseline NIHSS varies. In the BEST trial, the baseline NIHSS in the MT group was 32 (Liu et al., 2020), the BASICS trial was 21 (Langezaal et al., 2021), and our study was 18. The subsequent pooled analysis of the two RCTs (Nogueira et al., 2021) and the latest published RCTs (BAOCHE study and ATTENTION study) (Jovin et al., 2022; Tao et al., 2022), which focused on patients with NIHSS ≥10, showed significant efficacy of MT in such a population (Jovin et al., 2022; Tao et al., 2022).

Beyond the identified predictors mentioned above, our study had some interesting findings. First, as revealed by many studies (Alemseged et al., 2019; Alexandre et al., 2021; Bouslama et al., 2017; Kwak & Park, 2020; Mokin et al., 2016; Szmygin et al., 2021; Wu et al., 2021), our study confirmed that the outcome of MT in BAO patients was not influenced by age. This is very different from the intravenous thrombolytic therapy, in which the older age is an independent risk factor for poor outcomes (Amitrano et al., 2016; Schonewille et al., 2009). This finding suggests that in BAO, age may not be a strict exclusion criterion for MT.

Second, we did not prove the OTR time and recanalization status affected the outcome. In univariable analysis, we specifically divided the process times into OTE, OTI, OTP, OTR, and PTR. However, none of them were statistically significant. The same negative result was reported by Alexandre et al. (2021), Guillaume et al. (2019), and Kwak and Park (2020), but not by Mokin et al. (2016) and Wu et al. (2021). Further analysis revealed that in the studies with negative result (Alexandre et al., 2021; Guillaume et al., 2019; Kwak & Park, 2020), the OTR were all less than 400 min. It was remarkably shorter than the studies with positive results in which OTR were all over 600 min. It indicated that quick action to reduce OTR is critical for patients with a longer onset time. Besides OTR, the importance of recanalization status was also controversial. TICI score was the most common tool to evaluate the recanalization status. Several studies demonstrated TICI 2b‐3 was the predictor of favorable outcome (Bouslama et al., 2017; Mokin et al., 2016; Wu et al., 2021), while others did not (Alexandre et al., 2021; Kwak & Park, 2020; Wu et al., 2021). This discrepancy is probably because the ischemic tolerance differs between the ACS and PCS. The functional outcome of BAO seemed more dependent on circulation collateral than recanalization status (Kwak & Park, 2020). In addition, Wu et al. (2021) revealed that TICI 2c/3 but not 2b‐3 influenced the outcome. Kwak and Park (2020) confirmed this conclusion and showed TICI 3 tended to be more frequent in the group with favorable outcome. It indicated that, compared with ACS, a complete reperfusion status is essential to achieve a favorable outcome in BAO. Besides, we have noted that the recanalization rate (TICI 2b‐3) was similar between favorable (80.4%) and unfavorable (82.6%) outcome groups (*p* = .433). So, the futile recanalization may be higher in the unfavorable group. Although the recanalization rate was not included in our final nomogram, futile recanalization was still the risk of poor outcome.

In summary, similar to Alexandre et al. (2021), our study revealed that the clinical outcome of MT in BAO was mainly influenced by stroke severity (previous stroke, pc‐ASPECTS score, and NIHSS score). We presumed that with the development of MT techniques and devices, the traditional prognostic factors in the medical treatment of stroke, such as gender, glucose, blood pressure, and comorbidities (Harvey, 2015), were no longer crucial for MT. Besides, our study did not confirm that procedure‐related factors (procedure time intervals and recanalization rate) affect the outcome. As the latest RCTs revealed a significantly favorable outcome in patients with NIHSS ≥10 (Jovin et al., 2022; Tao et al., 2022), more detailed inclusion criteria may be needed to identify the potential beneficiaries for MT in BAO.

This study had several limitations. First, due to a low incidence rate, like most other studies of BAO, the small sample size could introduce selection bias and limit the generalizability of the findings. Second, the patients were enrolled from October 2016 to June 2021. During the 5 years, the revascularization device was upgraded, and the neurointerventionalists’ operative skills were improved. These factors may influence the MT outcome, and the small sample will further enlarge the effect. Third, data about collateral circulation status was missing and not included in the analysis.

## 5 CONCLUSIONS

In this study, dynamic and visual nomograms were constructed and validated for BAO patients treated with MT, which provided precise and online predictions of the risk of unfavorable outcomes at 90 days. The preoperative model may assist clinicians in selecting eligible MT patients and the postoperative model may facilitate individualized post‐stroke management.

## AUTHOR CONTRIBUTIONS

Xiding Pan: Study design, data collection & writing the initial draft. Shiteng Lin: Data analysis & writing the initial draft. Liang Xiang: Data collection. Feng Zhou: Data collection. Mengyi Xu: Data collection. Jie Qiong: Data collection. ZhiHong Zhao: Reverse the manuscript. Chen Chen: Reverse the manuscript. Junshan Zhou: Study design & supervised the study. Jianjun Zou: Study design & supervised the study.

## CONFLICT OF INTEREST STATEMENT

The authors declared that there is no conflict of interest.

### INSTITUTIONAL REVIEW BOARD STATEMENT

The study was conducted according to the guidelines of the Declaration of Helsinki. The studies involving human participants were reviewed and approved by the Ethics Committee of Nanjing First Hospital and People's Hospital of Hunan Province (approval number KY20130424‐01).

### INFORMED CONSENT STATEMENT

Informed consent was obtained from all subjects involved in the study.

### PEER REVIEW

The peer review history for this article is available at https://publons.com/publon/10.1002/brb3.3297

## Supporting information

Supplemental Figure 1. The example diagram of preoperative nomogram model on the web page.

Supplemental Figure 2. The example diagram of postoperative nomogram model on the web page.

Supplemental Table 1. Demographic and clinical characteristics comparing patients with favorable versus unfavorable outcome.

## Data Availability

The data are available on request from the corresponding author.
