# Supplementary figures and images for: Dynamic and visual nomograms to online predict unfavorable outcome of mechanical thrombectomy for acute basilar artery occlusion
Source: Brain Behav. 2023 Nov 13;13(12):e3297. doi: 10.1002/brb3.3297 (PMC10726912; doi:10.1002/brb3.3297)

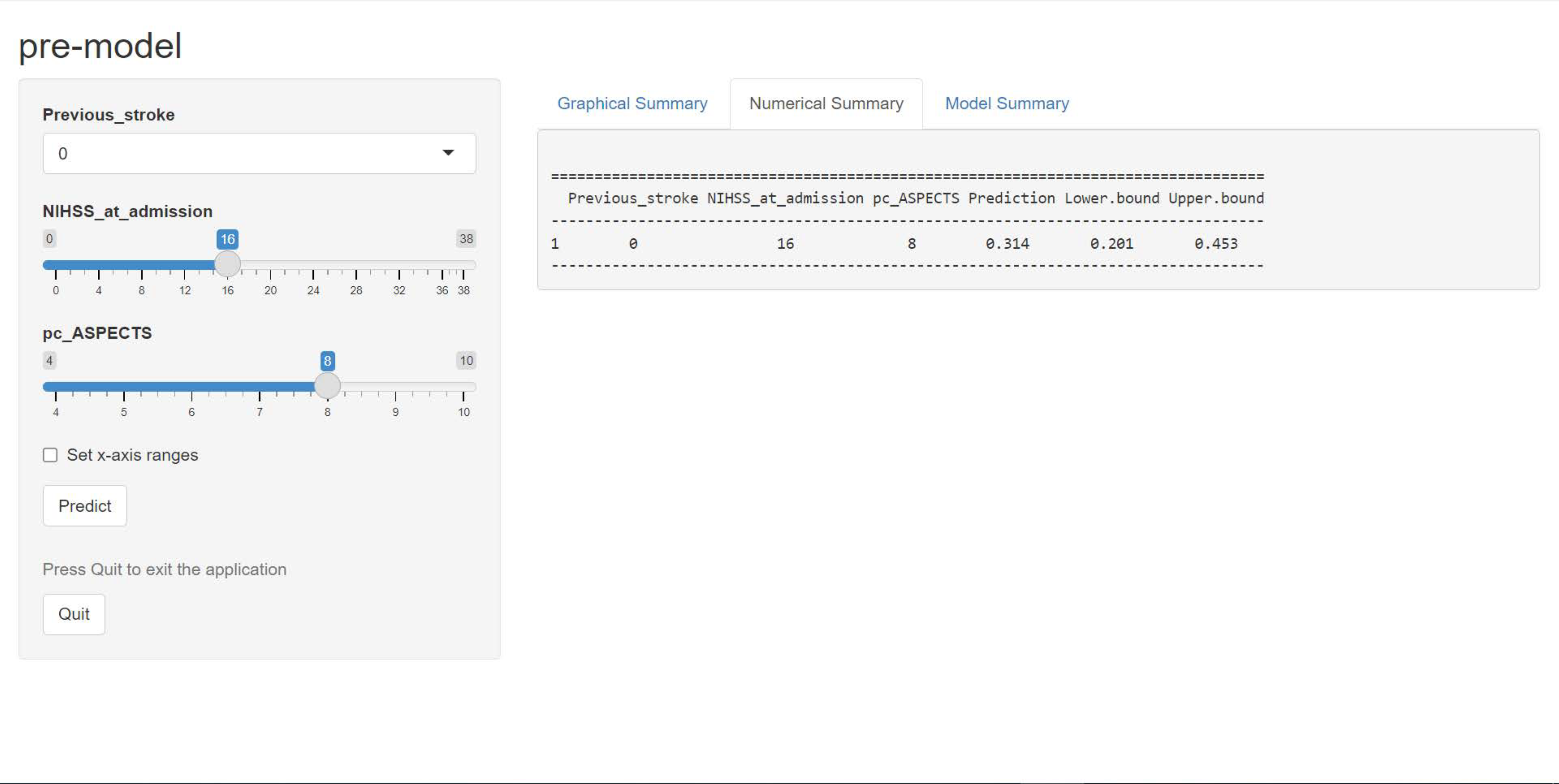

Supplement: Supplementary file 1 — Supplemental Figure 1. The example diagram of preoperative nomogram model on the web page. [file BRB3-13-e3297-s001.Tiff]

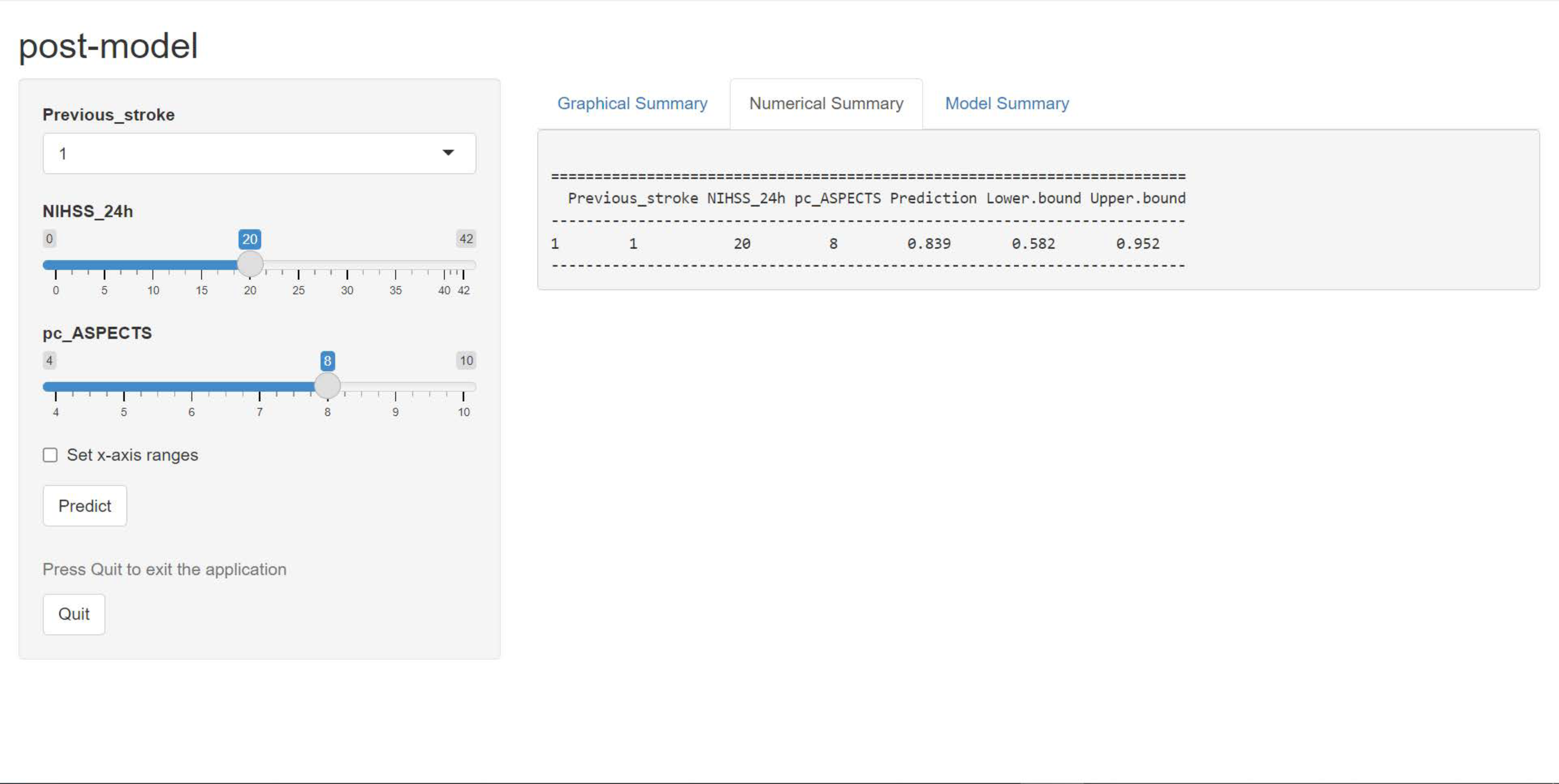

Supplement: Supplementary file 2 — Supplemental Figure 2. The example diagram of postoperative nomogram model on the web page. [file BRB3-13-e3297-s002.Tiff]
