# Supplementary material for: Dynamic and visual nomograms to online predict unfavorable outcome of mechanical thrombectomy for acute basilar artery occlusion
Source: Brain Behav. 2023 Nov 13;13(12):e3297. doi: 10.1002/brb3.3297 (PMC10726912; doi:10.1002/brb3.3297)
Supplement: Supplementary file 3 — Supplemental Table 1. Demographic and clinical characteristics comparing patients with favorable versus unfavorable outcome. [file BRB3-13-e3297-s003.DOCX]

**Supplemental Table 1.** Demographic and clinical characteristics comparing patients with favorable versus unfavorable outcome

|  | **Total**  **(n=97)** | **Unfavorable outcome (n=46) (mRS: 4-6)** | **Favorable outcome (n=51) (mRS: 0-3)** | **p-value** |
| --- | --- | --- | --- | --- |
| Demographic |  |  |  |  |
| Age, years, median (IQR) | 68 (61-76) | 67.50 (62.75-78) | 69.00 (58.00-75.00) | 0.237 |
| Male sex, n (%) | 75 (77.3%) | 36 (78.3%) | 39 (76.5%) | 0.833 |
| BMI, kg/m^2^, median (IQR) | 24.82 (22.36-26.49) | 25.02 (22.02-26.38) | 24.82 (22.49-27.06) | 0.806 |
| Education, years, n (%) |  |  |  | 0.521 |
| 0-6 | 45 (46.4%) | 25 (54.3%) | 20 (39.2%) |  |
| 6-9 | 23 (23.7%) | 9 (19.6%) | 14 (27.5%) |  |
| 9-12 | 17 (17.5%) | 7 (15.2%) | 10 (19.6%) |  |
| > 12 | 12 (12.4%) | 5 (10.9%) | 7 (13.7%) |  |
| Comorbidities |  |  |  |  |
| Hypertension, n (%) | 76 (78.4%) | 37 (80.4%) | 39 (76.5%) | 0.636 |
| Diabetes mellitus, n (%) | 28 (28.9%) | 15 (32.6%) | 13 (25.5%) | 0.440 |
| Dyslipidemia, n (%) | 34 (35.1%) | 14 (30.4%) | 20 (39.2%) | 0.365 |
| Coronary artery disease, n (%) | 12 (12.4%) | 4 (8.7%) | 8 (15.7%) | 0.296 |
| Atrial fibrillation, n (%) | 17 (17.5%) | 9 (19.6%) | 8 (15.7%) | 0.616 |
| Previous stroke, n (%) | 24 (24.7%) | 19 (41.3%) | 5 (9.8%) | <0.001 |
| Smoking |  |  |  | 0.564 |
| Never smoked (%) | 43 (44.3%) | 23 (50.0%) | 20 (39.2%) |  |
| Former smoker (%) | 12 (12.4%) | 5 (10.9%) | 7 (13.7%) |  |
| Current smoker (%) | 42 (43.3%) | 18 (39.1%) | 24 (47.1%) |  |
| Drinking |  |  |  | 0.126 |
| Never drank (%) | 62 (63.9%) | 33 (71.7%) | 29 (56.9%) |  |
| Former drinker (%) | 3 (3.1%) | 0 (0.0%) | 3 (5.9%) |  |
| Current drinker (%) | 32 (33.0%) | 13 (28.3%) | 19 (37.3%) |  |
| TOAST classification |  |  |  |  |
| Large-artery atherosclerosis (%) | 65 (67.0%) | 29 (63.0%) | 36 (70.6%) | 0.430 |
| Cardioembolism (%) | 27 (27.8%) | 13 (28.3%) | 14 (27.5%) | 0.929 |
| Lacunar | 1 (1.0%) | 1 (2.2%) | 0 (0.0%) | 0.474 |
| Other determined etiology (%) | 1 (1.0%) | 1 (2.2%) | 0 (0.0%) | 0.474 |
| Undetermined etiology (%) | 3 (3.1%) | 2 (4.3%) | 1 (2.0%) | 0.602 |
| Baseline data |  |  |  |  |
| Onset after waking up (%) | 13 (13.4%) | 7 (15.2%) | 6 (11.8%) | 0.618 |
| NIHSS at admission, median (IQR) | 18 (10-31) | 28 (16-35) | 13 (5-22) | <0.001 |
| PC-ASPECTS, median (IQR) | 8 (7-10) | 8 (6-9) | 9 (8-10) | 0.002 |
| Intravenous thrombolysis (%) | 30 (30.9%) | 12 (26.1%) | 18 (35.3%) | 0.327 |
| SBP, mmHg, median (IQR) | 140 (128-159) | 140 (130-156) | 140 (123-159) | 0.960 |
| Platelet count, 10^9^/L, median (IQR) | 194 (151-240) | 192 (151-236) | 196 (151-244) | 0.492 |
| Uric acid, median (IQR) | 314 (234-399) | 306 (238-397) | 321 (224-399) | 0.991 |
| INR, median (IQR) | 0.96 (0.93-1.01) | 0.98 (0.95-1.02) | 0.95 (0.91-1.00) | 0.020 |
| FBG, mmol/L, median (IQR) | 6.75 (5.69-8.12) | 7.25 (6.17-8.69) | 6.21 (5.43-7.58) | 0.016 |
| TC, mmol/L, median (IQR) | 4.65 (3.93-5.31) | 4.52 (3.91-5.21) | 4.72 (4.01-5.40) | 0.439 |
| TG, mmol/L, median (IQR) | 1.15 (0.80-1.68) | 1.07 (0.78-1.54) | 1.16 (0.84-1.75) | 0.549 |
| HDL, mmol/L, median (IQR) | 1.04 (0.87-1.25) | 1.05 (0.91-1.20) | 1.03 (0.80-1.25) | 0.382 |
| LDL, mmol/L, median (IQR) | 2.92 (2.40-3.42) | 2.75 (2.37-3.21) | 3.08 (2.45-3.44) | 0.228 |
| Prior IV tPA (%) | 32 (33.0%) | 12 (26.1%) | 20 (39.2%) | 0.170 |
| Intraoperative and postoperative variables |  |  |  |  |
| sICH (%) | 8 (8.2%) | 7 (15.2%) | 1 (2.0%) | 0.025 |
| NIHSS at 24h, median (IQR) | 16 (4-33) | 32 (21-36) | 5 (2-14) | <0.001 |
| TICI 2b-3 (%) | 79 (81.4%) | 38 (82.6%) | 41 (80.4%) | 0.779 |
| Number of retrievals, median (IQR) | 2 (1-2) | 1 (1-2) | 2 (1-2) | 0.925 |
| Postoperative complications |  |  |  |  |
| Respiratory infections (%) | 71 (73.2%) | 36 (78.3%) | 35 (68.6%) | 0.285 |
| Secondary epilepsy (%) | 2 (2.1%) | 2 (4.3%) | 0 (0.0%) | 0.132 |
| Gastrointestinal bleeding (%) | 8 (8.2%) | 6 (13.0%) | 2 (3.9%) | 0.145 |
| Electrolyte disorders (%) | 77 (79.4%) | 42 (91.3%) | 35 (68.6%) | 0.006 |
| Blood pressure variability parameters |  |  |  |  |
| SBP |  |  |  |  |
| SD, median (IQR) | 13.32 (9.02-19.08) | 13.35 (8.40-19.50) | 13.32(9.76-18.88) | 0.729 |
| CV, median (IQR) | 10.27 (7.07-14.72) | 10.43 (6.93-15.62) | 10.27 (7.06-14.41) | 0.986 |
| DBP |  |  |  |  |
| SD, median (IQR) | 7.86 (5.37-12.75) | 9.17 (5.76-12.99) | 7.35 (5.17-11.64) | 0.287 |
| CV, median (IQR) | 10.98 (6.64-16.78) | 12.18 (7.47-17.04) | 9.79 (6.19-16.40) | 0.193 |
| Process times (min) |  |  |  |  |
| OTE, median (IQR) | 180 (93-345) | 178 (75-370) | 195 (115-330) | 0.527 |
| OTI, median (IQR) | 260 (147-402) | 219 (136-394) | 285 (150-415) | 0.418 |
| OTP, median (IQR) | 333 (230-482) | 282 (196-446) | 350 (235-510) | 0.291 |
| OTR, median (IQR) | 400 (300-559) | 367 (287-541) | 420 (340-563) | 0.189 |
| PTR, median (IQR) | 80 (56-114) | 86 (55-121) | 75 (60-105) | 0.382 |

IQR, interquartile range; BMI, body mass index; mRS, modified Rankin Scale; NIHSS, National Institutes of Health Stroke Scale; pc-ASPECTS, posterior circulation Alberta Stroke Program Early Computed Tomography Score; SBP, systolic blood pressure; DBP, diastolic blood pressure; INR, international normalized ratio; FBG, fasting blood glucose; TC, total cholesterol; TG, triglyceride; HDL, high-density lipoprotein; LDL, low-density lipoprotein; IV tPA, intravenous thrombolysis; ICH, symptomatic intracranial hemorrhage; TICI, The Thrombolysis in Cerebral Infarction score; SD, standard deviation; CV, coefficient of variation; OTE, onset to emergency; OTI, onset to image; OTP, onset to puncture; OTR, onset to recanalization; PTR, puncture to recanalization. Data are given as n (%) or median (interquartile range). ^†^Variables selected by the least absolute selection and shrinkage operator regression.
